# Sex Steroids and the Shaping of the Peripubertal Brain: The Sexual-Dimorphic Set-Up of Adult Neurogenesis

**DOI:** 10.3390/ijms22157984

**Published:** 2021-07-26

**Authors:** Sara Trova, Serena Bovetti, Sara Bonzano, Silvia De Marchis, Paolo Peretto

**Affiliations:** 1 Department of Life Sciences and Systems Biology (DBIOS), University of Torino, 10123 Turin, Italy; sara.trova@iit.it (S.T.); serena.bovetti@unito.it (S.B.); sara.bonzano@unito.it (S.B.); silvia.demarchis@unito.it (S.D.M.); 2 Neuroscience Institute Cavalieri Ottolenghi (NICO), Orbassano, 10043 Turin, Italy

**Keywords:** puberty, gonadal hormones, adult neurogenesis, sexual dimorphism, pheromones

## Abstract

Steroid hormones represent an amazing class of molecules that play pleiotropic roles in vertebrates. In mammals, during postnatal development, sex steroids significantly influence the organization of sexually dimorphic neural circuits underlying behaviors critical for survival, such as the reproductive one. During the last decades, multiple studies have shown that many cortical and subcortical brain regions undergo sex steroid-dependent structural organization around puberty, a critical stage of life characterized by high sensitivity to external stimuli and a profound structural and functional remodeling of the organism. Here, we first give an overview of current data on how sex steroids shape the peripubertal brain by regulating neuroplasticity mechanisms. Then, we focus on adult neurogenesis, a striking form of persistent structural plasticity involved in the control of social behaviors and regulated by a fine-tuned integration of external and internal cues. We discuss recent data supporting that the sex steroid-dependent peripubertal organization of neural circuits involves a sexually dimorphic set-up of adult neurogenesis that in turn could be relevant for sex-specific reproductive behaviors.

## 1. Introduction

In vertebrates, postnatal development involves an important phase of brain circuitry organization/refinement that is critical for reaching a behavioral repertoire—including reproductive behavior—typical of fully mature animals. The duration and modalities through which this process takes place depend on the evolutionary history of each taxon. Nevertheless, a commonly conserved trait among several animal taxa is that every experience occurring during the early postnatal period can permanently shape/adapt the genetic basis of the nervous system architecture and thus its neural networks [1]. This remarkable feature of the nervous system, known as neural plasticity, implies the ability of the neural tissue to undergo adaptive modifications under the influence of both external and internal stimuli and, in parallel, the capability to integrate such stimuli. Among the internal stimuli that are able to significantly model the brain circuits postnatally, gonadal steroid hormones play a primary and peculiar role. Steroid hormones, together with the enzymes and receptors involved in their biosynthesis and biological responses, are universal bio-regulators active in both multi- and unicellular organisms, representing an amazing example of molecular evolutionary parsimony [2]. In mammals, several studies have demonstrated that gonadal steroids play a critical role in organizing the brain in a sex-specific way during perinatal life [3,4,5,6,7]. Afterwards, starting around puberty, they activate sexually dimorphic behaviors, such as the reproductive one, by acting on the same circuitries they had previously shaped [8,9]. The activation of such circuits requires integration with external sexual cues (i.e., pheromones) processed by the olfactory system [10,11]. Notably, pheromones released by conspecifics are often gonadal steroid derivatives, whose secretion abundance and efficacy strongly depend on the circulating levels of gonadal hormones in both the donor and receiver [5,12,13,14].

During the last years, a further level of control exerted by gonadal steroids on the activity of the mammalian brain circuits underlying sex-related behaviors has been proposed, through modulation of adult neurogenesis [15,16,17]. Adult neurogenesis is an extreme form of neural plasticity that occurs in two regions of the adult mammalian brain (i.e., dentate gyrus of the hippocampus and olfactory bulb, OB), and implies genesis and functional integration of new neurons throughout life [18,19,20]. In the OB of rodents, both in the main (MOB) and accessory (AOB) regions, newborn cells are inhibitory interneurons that through their modulatory role on the OB output influence downstream circuits involved in the control of the reproductive behavior [21,22,23]. Indeed, impaired adult neurogenesis has been shown to affect crucial sex-specific behaviors, such as mate choice and mate recognition, as well as the opposite sex-preference [17,24,25]. Moreover, in the AOB of female mice, exposure to opposite-sex pheromones [14,26], which triggers reproductive behavior, enhances the survival of newborn neurons [14,24] and in parallel promotes gonadal hormones release [27]. Notably, the steroid gonadal hormones, but also amino acidic-derived gonadal hormones (e.g., prolactin and gonadotropins), modulate the proliferation of progenitor cells and/or the survival of newly-generated neurons, with different outcomes depending on age, sex, physiological state, hormonal milieu and species [16,28]. Taken together, these data support the idea that gonadal hormones can modulate the reproductive behavior, not only through directly activating underlying neural networks but also through regulating genesis and integrating new inhibitory interneurons in the OB circuits processing pheromonal cues. In this review, we focus on the role of gonadal hormones during peripubertal life or peripuberty, which includes puberty and adolescence (i.e., transitional stage of life from puberty to adulthood) [29]. First, we discuss data indicating that in some cortical and subcortical neural circuits the sex steroid-dependent organizational phase extends well into peripuberty by modulating neural plasticity mechanisms. Then, we focus on the role of peripubertal gonadal hormones in shaping adult neurogenesis in a sexually dimorphic way. To this aim, we present recent results mostly obtained in the GnRH::cre;Dicer^loxP/loxP^ mice, a model of impaired peripubertal secretion of gonadal hormones [30]. In these animals, the secretion of the gonadotropin-releasing hormone (GnRH), which is key to drive the onset of puberty, is progressively and irreversibly lost during the infantile period, resulting in sever hypogonadism and sterility in adulthood [31].

## 2. Sex Steroid-Dependent Refinement of Brain Circuits and Behaviors during Peripuberty

The peripubertal life is a rather complex transitional phase marked by profound physiological, physical and behavioral changes aimed at achieving adult life cognitive and reproductive competencies [29]. This developmental phase is triggered by the integration of internal cues, such as hormones and energy resources, with external factors, including the photo-period, but also with signals of different nature (e.g., chemical, visual, acoustic) coming from conspecifics [32,33,34]. One key molecular activator of puberty is GnRH, which is produced by a small and highly conserved population of neurons (i.e., GnRH neuronal system, composed of a few thousand neurons in vertebrates), mostly located in the hypothalamus [35,36]. Evidence accumulated in the last decades indicated that in mammals the pubertal onset is timed by an increase of pulsatile GnRH secretion that is triggered by the activity of the so called “GnRH pulse generator”. This pulse generator includes the kisspeptin/neurokinin B/dynorphin A neurons located in the hypothalamic arcuate nucleus [37], whose activity is tightly controlled by steroid-dependent (rodents and sheep) or steroid-independent (primates) mechanisms (reviewed in [38]). Therefore, GnRH neurons work as a neuroendocrine biological clock orchestrating the activity of the hypothalamic pituitary gonadal (HPG) axis and thus acting as a driving force for the gonadal maturation and the release of sex steroids. Accordingly, the circulating levels of these hormones significantly rise at the onset of puberty [39,40,41,42], and in parallel, sex-specific morphological traits and behaviors start to appear [34].

According to the classic view, the expression of dimorphic behaviors during peripubertal life is granted by the hormone-induced activation of neural circuits whose organization relies on the same hormones perinatally [43,44,45]. Indeed, from the seminal work of Phoenix from 1959 onward, the common leitmotif is that a transient perinatal rise of testosterone is required to masculinize and defeminize male neural and peripheral tissues. By contrast, the absence of testosterone determines a feminine neural phenotype [46]. Interestingly, this structuring activity occurs through an early critical period of maximal sensitivity to sex steroids [3], which irreversibly shapes/organizes several neural circuits in a sexually dependent way [47].

**Figure 1:**
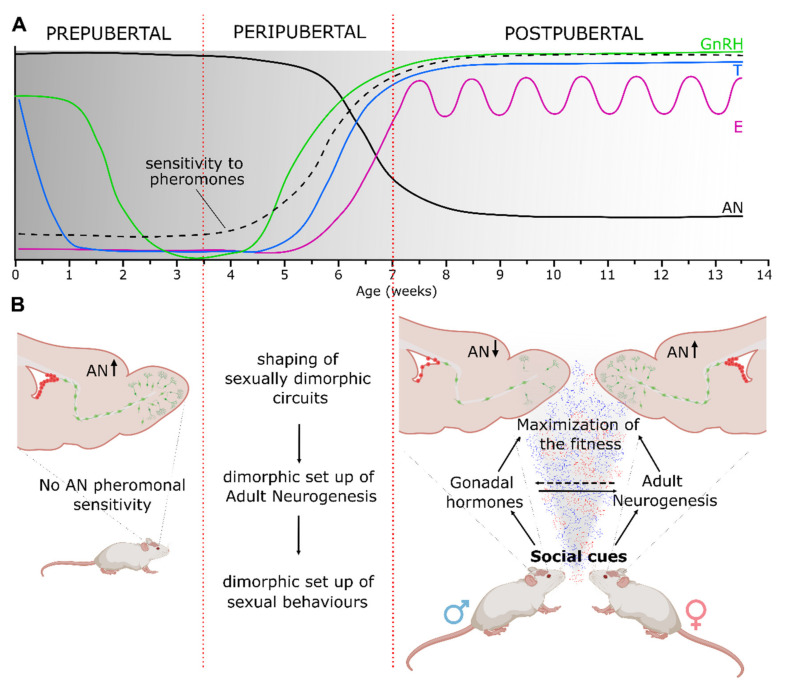
Sex hormones and adult neurogenesis in the mouse brain. In (**A**) is represented the brain sensitivity to sex hormones and pheromones and the time course of endogenous secretions and adult neurogenesis from prepubertal to postpubertal life. According to the “two-stage model” proposed in Schulz and Sisk (2016), the gray to white nuance in the picture illustrates the progressive postnatal reduction of structural brain sensitivity to sex steroids, which peaks during the early prepubertal life (0–3.5 weeks) and peripuberty (3.5–7 weeks), in coincidence with GnRH, testosterone and estradiol release (solid-colored lines). The onset of puberty is triggered by GnRH increase secretion, which results in a rise of circulating sex steroids. In the peripubertal period, adult neurogenesis (solid black line) halves, coinciding with the increase in sex steroids, and in females starts to be modulated by male pheromones (dotted black line). In (**B**) is depicted a representation of adult neurogenesis in the SVZ/OB region during postnatal life and its pheromonal-dependent modulation in females. In the prepubertal period, the level of AN is elevated (green and red cells) and it does not change upon exposure to opposite-sex pheromones. Starting from the late peripubertal period male pheromones stimulate AN and secretion of gonadal hormones, which reciprocally interact to optimize reproduction. Note that peripubertal life is regarded as an important phase of structural and functional hormone-dependent refinement of sexually dimorphic circuits, which includes a set-up of adult neurogenesis in both male and female mice. Abbreviations: AN, adult neurogenesis; E, estradiol; GnRH, gonadotropin-releasing hormones; T, testosterone; SVZ/OB, subventricular zone/olfactory bulb.

During the last decades, the issue of sexual brain organization and activation has been progressively fueled by novel data revealing a greater degree of complexity both at the molecular level and in terms of temporal windows of sensitivity to hormones [48,49,50]. For instance, it has been shown that, in addition to a primary role of testosterone, its metabolites and the ovarian hormones have an active role in the set-up of several sex-specific neural brain circuits and behaviors. Moreover, it is now clear that in both rodents and humans, the sex steroid-dependent brain organization not only involves circuits underlying sexual behavior but also involves those related to cognitive and executive functions, which typically emerge only after the onset of puberty and that show sex-specific features [51]. Consistently, a growing body of evidence is revealing peripuberty as a second period of brain sensitivity to gonadal steroids, functionally related to a final refinement of neural substrates underlying some social and cognitive behaviors [52]. In particular, a significant contribution to this issue came from studies in the Syrian hamster [45,52]. In this species, experimental alteration of the circulating level of gonadal steroids through either gonadectomy or steroid treatments at specific time windows from perinatal life to adulthood, revealed the need for peripubertal steroids to sustain adult sex-related social and reproductive responses, such as lordosis in females or flank marking, intromission and mounting in males [43,53,54]. The authors of these studies introduced a two-stage model of postnatal brain and behavioral development [52]. In this model, rather than considering two distinct periods of brain sensitivity to steroid hormones, the presence of only one extended sensitivity window from early postnatal life to the postpubertal stage was suggested as being more likely. Within this responsive period, male and female sex hormones would shape the brain accordingly in response to their fluctuations. This model brings forth the idea of a double organizational stage for testosterone in males, which shows two secretory peaks, whereas only one estradiol-dependent peak is restricted to puberty in females (see Figure 1A). Although the two-stage model of brain sensitivity needs further confirmation, the importance of peripubertal sex steroids to the final shaping of sexually dimorphic circuits driving adult-like (activational) responses to gonadal hormones and environmental social cues has been confirmed in other mammalian species [48,55,56] including humans (e.g., spatial ability, [57,58]).

## 3. Mechanisms Underlying the Sex Steroid-Dependent Refinement of the Brain at Peripubertal Ages

An additional key question concerns the way through which sex steroids remodel neural circuits and behaviors during puberty. It has been shown that the level of male and female sex steroids at puberty does influence several aspects related to neural plasticity depending on sex, brain domain and associated neural networks (for review see [45,53]), and recent evidence has shown a coincidence between the rise of sex steroids and the enhancement in experience-dependent plasticity at puberty [56,57,59,60]. This steroid-dependent regulatory activity on neural plasticity involves modulation in the expression of neurotransmitters and neurotrophic factors, dendritic spine formation and pruning, myelination and regulation of cell number [43,48,50,51,61,62,63,64,65].

As previously mentioned, neural plasticity around puberty, especially for what concerns some cortical regions (e.g., prefrontal cortex), is important, rather than for basic developmental processes (e.g., sensorimotor processing) for allowing the set-up of cognitive and executive functions, which continue to evolve during this stage of life [50,64,65]. The opening and closing of critical periods in cortical regions rely on a fine-tuned balancing between excitation and inhibition, and the maturation of the GABAergic inhibitory neurotransmission plays a primary role in this mechanism [66,67]. Experimental manipulation of gonadal hormones around puberty in female mice provided clear evidence that ovarian steroids promote the development of inhibitory neurotransmission in the prefrontal cortex [55]. Specifically, the inhibitory neurotransmission that increases during peripubertal development was blocked by prepubertal but not postpubertal gonadectomy. Moreover, anticipating the onset of puberty through hormone treatments during the prepubertal phase resulted in advanced maturation of both the inhibitory system and the expression of a behavioral phenotype typical of adulthood, indicating a link between the rise of gonadal hormones at puberty and neurobehavioral development [47]. In addition, this effect was not found in the somatosensory cortex, showing a region-specific contribution of gonadal steroids in modulating critical periods of plasticity during peripuberty. Similarly, in another study, it has been shown that in the dorsal hippocampus of female mice, starting from puberty, there is a gradual increase expression of the GABAergic interneuron marker parvalbumin (PV) that correlates with circulating 17β-estradiol levels [54]. Prepubertal ovariectomy impairs PV expression (via selective downregulation of estrogen receptor alpha in PV cells), which is recovered by simultaneous treatment with 17β-estradiol. Notably, the same set of analyses (including prepubertal gonadectomy) performed in males throughout the same temporal window did not show any correlation between male gonadal steroids and modulation of PV expression in the hippocampus [54], indicating that brain sensitivity mediated by sex steroids around puberty is region- and sex-specific. 

Steroid-dependent peripubertal plastic remodeling also occurs in several subcortical domains processing salient social cues and characterized by sexual dimorphism [28,51]. By systemic injections of the cell birth-date marker bromodeoxyuridine before and during early and mid-puberty in male and female rats, Ahmed and collaborators [68] quantified the number of newborn cells of three subcortical sexually dimorphic nuclei involved in the control of reproductive behavior [10,69]: the anteroventral periventricular nucleus of the hypothalamus (AVPV), the sexually dimorphic nucleus of the preoptic area (SDN) and the medial amygdala. The AVPV is larger in females than in males, while the opposite is true for SDN and medial amygdala. The quantitative analysis indicated sex differences (i.e., AVPV showing a higher number of BrdU cells in females than in males and vice versa for the SDN and medial amygdala), independent of the timing of BrdU administrations. Notably, the removal of gonadal hormones before puberty selectively affected cell genesis. In particular, BrdU labeled cells decreased in the AVPV of females and not in males, whereas BrdU-positive cells were reduced in the SDN and medial amygdala only in males [44]. These data are consistent with the idea that sex steroids act as agents of neural rearrangements around puberty, as also confirmed by subsequent studies (e.g., [1,70]), and disclose a postnatal neurogenic regulation likely involved in establishing sexually dimorphic features in specific neural circuits of either the male or female brain. In the following paragraphs, we focus on a form of postnatal neurogenesis that persists throughout life (i.e., adult neurogenesis) and represents a unique process of structural plasticity, which is regulated by gonadal hormones and involved in the modulation of sensory cues triggering reproduction.

## 4. The Interplay among Pheromones, Hormones and Adult Neurogenesis in the Regulation of Reproductive Activities

The demonstration that adult neurogenesis in rodents involves integration of newborn neurons in the AOB [71,72,73] raised the hypothesis that this process could be linked to the regulation of social behaviors, including reproductive behaviors (see in example for review [51]). Indeed, the AOB represents the first brain station of the vomeronasal system, a subcortical pathway involved in the integration of sensory cues eliciting social behaviors [74,75,76]. In particular, AOB newborn cells are GABAergic interneurons that play a crucial role in the modulation of sensory outputs to the vomeronasal downstream nuclei [23,40,77]. One of the first pieces of evidence linking adult neurogenesis in the AOB with reproduction came from the experiments in which sexually mature female mice were exposed to male pheromones [25]. By BrdU birth-dating analyses, it was shown that such experience enhances the survival of newborn neurons while they are integrating within the AOB neural circuits [14,16,25]. Notably, this pro-survival effect appeared to be sex specific since exposure of male mice to male pheromones did not promote the survival of new neurons in the AOB [14,23]. In parallel, other studies showed that olfactory perception of male pheromones in females also stimulates proliferation of neuronal-committed progenitors in the germinative regions of both the subventricular zone (SVZ) of the lateral ventricles and the subgranular zone of the hippocampal dentate gyrus [23,78,79,80]. This enhancement in cell proliferation was found to be mostly mediated by the circulating level of HPG axis hormones (gonadotropins, prolactin, gonadal hormones), which is actually influenced by a pheromonal-dependent activation of the main and accessory olfactory pathways [27,79]. In addition, hormonal-dependent proliferation of OB progenitors in female mice was also enhanced by certain physiological states, such as pregnancy and lactation [80]. Thus, this first set of data clearly showed how the modulation of adult neurogenesis results from integration of salient environmental social stimuli (i.e., pheromones) and hormones. Eventually, by using diverse approaches to ablate OB neurogenesis, it was demonstrated that adult neurogenesis is directly involved in mediating social behaviors [23,59,81]. Indeed, decreasing the number of newborn neurons integrating in the OB circuits negatively affected some female mice behaviors related to reproduction, such as mate choice [82] and mate pheromonal imprinting [23]. In particular, this latter behavior consists of a neuroendocrine reflex that allows female mice to avoid the pregnancy block (known as the “Bruce Effect”) [26] when exposed to the mating male [83]. Specifically, when a recently mated female perceives unfamiliar male olfactory cues, it undergoes pregnancy block due to pheromonal-dependent activation of the vomeronasal pathway that prevents the adeno-hypophyseal release of prolactin (luteotropic in mice), thus resulting in an abortion [84]. By contrast, a mate’s pheromones do not stimulate this reflex, since, during familiarization and coupling, females imprint the AOB neural circuits on mating male odors [85]. Such form of memory efficiently blocks the vomeronasal-dependent signal that drives to pregnancy block and requires newborn integrated interneurons in the AOB [24]. Thus, the mate pheromonal imprinting is a clear example that illustrates how optimization of reproductive-related behaviors requires a fine-tuned integration of pheromonal stimuli, adult neurogenesis and HPG axis secretory activity.

## 5. Role of Hormones on the Sexually Dimorphic Set-Up of Adult Neurogenesis at Puberty

As discussed above, during adulthood, HPG axis hormones and neurogenesis cooperate to support appropriate responses to social stimuli. This relationship not only involves adult neurogenesis in the olfactory neurogenic niche but also implicates the hippocampal neurogenic system, which can be differentially modulated in response to diverse factors/conditions (see for exhaustive review [79,86,87]).

**Figure 2:**
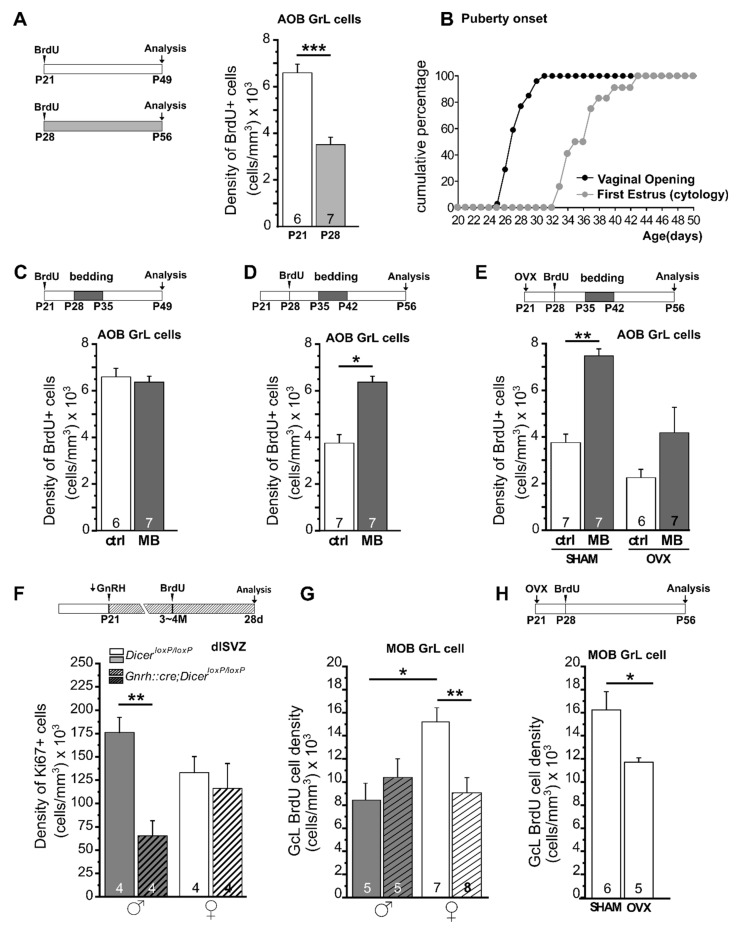
Time course and modulation of adult neurogenesis during peripubertal life and adulthood. (**A**) Injections of the cell birth marker BrdU before puberty (P21) results in double the number of 28-day-old newborn granule cell (GC) neurons in the AOB when compared with BrdU treatments performed after the onset of puberty (at P28) (unpaired Student’s *t*-test, P21 vs. P28, *p* = 0.0004), indicating a sharp decrease of adult neurogenesis during peripubertal life. (**B**) Graph showing the onset of puberty in female mice determined by examination of vaginal opening and occurrence of the first estrus. (**C**,**D**) Daily exposure of female mice to pheromones (male soiled bedding, MB) increases GC survival only after the onset of the first estrus cycle (P35–P42). Bedding stimulations were timed during the 2nd week after GC genesis and their survival evaluated 28 dpi (unpaired Student’s *t*-test, P28–P35 ctrl vs. MB, *p* = 0.669, P35–P42 ctrl vs. MB, ⇤ *p* = 0.016) (see Oboti et al. 2017 for major details). (**E**) Bedding exposure in ovariectomized (OVX) prepubertal females (P21) does not modulate GC survival as significantly as in SHAM-operated animals (two-way ANOVA, factors: ovariectomy, *F*(2,10) = 6.766, *p* = 0.017; stimulus, *F*(2,10)= 12.14, *p* = 0.002; interaction, *F*(2,10)= 0.520, *p* = 0.479; Tukey’s post hoc, SHAM ctrl vs. SHAM MB, ⇤ *p* = 0.045; OVX ctrl vs. OVX MB, *p* = 0.206). (**F**) In the GnRH::cre/Dicer^loxP/loxP^ mice, GnRH is significantly downregulated starting from P21. Adult neurogenesis in these animals was quantified by BrdU injections at 3–4 months of age. The density of progenitors cells (Ki67+ cells) in the dorsolateral subventricular zone (dlSVZ) is significantly reduced in males, whereas no differences were found in females. (**G**) By contrast, in the main olfactory bulb (MOB), only females showed a reduction in newborn cell survival (density of BrdU+ cells). Interestingly, wild-type females have a higher density of BrdU cells compared with wild-type males, indicating sexually dimorphic neurogenesis in this region. (**H**). Survival of 28-day-old newborn granule cells in the MOB of control females ovariectomized at P21 (when GnRH in GnRH::cre/Dicer^loxP/loxP^ mice is downregulated) and injected with BrdU at P28 shows significant reduction when compared with SHAM operated females (Student’s *t*-test, * *p* = 0.032), suggesting that the alteration of adult neurogenesis identified in GnRH::cre/Dicer^loxP/loxP^ females are mostly attributable to impaired sex steroids secretion (see the text and Trova et al. 2020 for major details). * *p* < 0.05; ** *p* < 0.01; *** *p* < 0.001. Abbreviations: ctrl, control; MB, male bedding; MOB, main olfactory bulb; AOB, accessory olfactory bulb; GrL, granule cell layer; OVX, ovariectomy; dpi, days post-injection. All graphs were taken or modified from [14,88].

Given the sex steroid-dependent remodeling of the brain at peripubertal ages by modulation of neural plasticity processes, one interesting question arises regarding the possible effects of such hormones in shaping adult neurogenesis at the onset of puberty, when the HPG axis starts to be activated and the circulating level of its secretions significantly rise. Interestingly, BrdU labelling of newborn neurons in female mice at specific days over the peripubertal period showed a sharp drop (of about 50%) of adult OB neurogenesis during the onset of puberty [14] (Figure 2A). Moreover, the pro-survival effect of male pheromones on AOB newborn neurons in females was only found after the occurrence of their first estrous and was found to be impaired when examined in females that were gonadectomized just before puberty [14,24] (Figure 2B–E). These results revealed a correlation among increased secretion of HPG-axis factors, quantitative regulation of adult neurogenesis and its sex-specific pheromonal-dependent modulation. To address the occurrence of a causal relationship among these factors, we examined the process of adult neurogenesis by exploiting different models of HPG-axis peripubertal inactivation in mice [14,88]. The analyses performed in the GnRH::Cre;Dicer^loxP/loxP^ mice [88] confirmed a direct influence of peripubertal hormones on the process of adult neurogenesis around puberty. In these animals, Dicer, an RNAse-III endonuclease essential for miRNA biogenesis [89], is selectively inactivated in GnRH neurons, resulting in a gradual loss of GnRH expression and secretion, which starts during the infantile period (p7–p20) and accelerates in the juvenile ages soon after weaning (p21~p35) [31] (Figure 2F). Thus, these mice lack the increased secretion of GnRH and downstream factors (e.g., gonadotropins and gonadal hormones) that trigger the onset of puberty, leading in turn to hypogonadism and sterility [31]. Notably, in these mice, the perinatal phase of sex brain circuitry organization remains preserved, considering both the GnRH-dependent and GnRH-independent (release of luteinizing hormone and testosterone) effects [90,91]. For these reasons, this model is particularly suitable for specifically investigating the impact of peripubertal alterations of HPG factors in shaping adult neural networks. A detailed study on adult neurogenesis in the OB and in the hippocampal neurogenic niches in adult male and female GnRH::Cre;Dicer^loxP/loxP^ mice, taking into account proliferation and neuronal commitment of progenitor cells, as well as the survival/integration of newborn neurons in the target regions [88], showed that impaired activity of the HPG axis around puberty impacts males and not females in the SVZ neurogenic niche (Figure 2F). By contrast, no significant differences were observed in the hippocampal neurogenic niche either in male or female GnRH::Cre;Dicer^loxP/loxP^ mice compared with controls. Sex- and region-specific modulation of adult neurogenesis in the GnRH::Cre;Dicer^loxP/loxP^ model was also identified when newborn cell survival was examined one month after BrdU injection, indicating a decrease in newborn neurons integrated in the OB regions, specifically in the MOB of female mice. Notably, this reduction abolished the occurrence of sexual dimorphism present in control animals (Figure 2G).

Overall, these data indicate that peripubertal HPG-axis secretions affect the process of adult neurogenesis selectively in the SVZ/OB neurogenic niche. Moreover, this activity appears critical to control sexually dimorphic characteristics of neurogenesis during adulthood, which are lost in absence of circulating peripubertal hormones. Although quite consistent with previous data in rodents showing that the level and type of circulating hormones actually influence adult neurogenesis [92,93,94,95,96,97,98], the results on the GnRH::Cre;Dicer^loxP/loxP^ mice became particularly interesting when compared with those from other models based on impairment of HPG function early during postnatal life or in adulthood (see in example [16,86]). Indeed, the responses obtained in GnRH::Cre;Dicer^loxP/loxP^ mice, in which HPG is compromised starting from the peripubertal life, appear very different, and in some cases opposite to those described in the other models, supporting findings showing that the loss of peripubertal hormones results in peculiar alterations of the adult neurogenic process. Finally, analysis of adult neurogenesis on standard females gonadectomized just before puberty (wherein GnRH and gonadotropins secretion is preserved) indicated that sex steroids, among the diverse HPG axis secreted factors, play a primary role in the peripubertal-dependent modulation of adult neurogenesis (Figure 2H). In conclusion, the control of adult neurogenesis by peripubertal hormones can be seen as an additional form of modulation of neural plasticity mechanisms through which gonadal hormones shape the organization of sexually dimorphic neural circuits at puberty, possibly influencing sex-related responses to social stimuli during adulthood. It is interesting to note that in the semaphorin7A knockout adult male mice [99], which are characterized by a low level of circulating testosterone during postnatal life [30], the exposures to male pheromones elicits “feminine-like responses” of both adult neurogenesis and neural activity in the vomeronasal circuits [17], clearly showing how sex-specific responses result from a fine-tuned balance/integration of sex steroids, adult neurogenesis and pheromones.

## 6. Conclusions

We have reported several pieces of evidence indicating that puberty is a sensitive period of life in which sex steroids sharply organize sex-dimorphic neural circuits and behavior. The brain shaping activity of sex-hormones involves several cortical and subcortical regions and occurs mainly through the modulation of neural plasticity mechanisms. Among those mechanisms, here we focused on adult neurogenesis, an extreme form of structural plasticity that occurs in a few brain areas, including the hippocampal dentate gyrus and the OB. We presented data indicating that the pubertal activation of the HPG axis secretory system (i.e., GnRH, gonadotropins, and gonadal hormones) is crucial to fine-tune the adult neurogenesis process selectively in the OB, a key sensory region in the processing of salient social cues. Here, the modulatory function of steroid hormones occurs differentially in males and females, indicating a critical role in the shaping of a sexually dimorphic neurogenic process in adulthood. Although further research is needed to better dissect the specific molecular mechanisms involved and to establish the precise behavioral consequences of this sex steroid peripubertal regulation of adult neurogenesis in the OB, the data collected so far point to the relevance of a sexually dimorphic set-up of adult neurogenesis in the context of sex-specific reproductive behaviors.

## Data Availability

Not applicable.
